# Corticosteroid insensitivity persists in the absence of STAT1 signaling in severe allergic airway inflammation

**DOI:** 10.1152/ajplung.00244.2021

**Published:** 2021-11-10

**Authors:** Brandon W. Lewis, Devine Jackson, Stephanie A. Amici, Joshua Walum, Manel Guessas, Sonia Guessas, Elise Coneglio, Akhila V. Boda, Mireia Guerau-de-Arellano, Mitchell H. Grayson, Rodney D. Britt

**Affiliations:** ^1^Center for Perinatal Research, The Abigail Wexner Research Institute at Nationwide Children’s Hospital, Columbus, Ohio; ^2^Center for Clinical and Translational Research, The Abigail Wexner Research Institute at Nationwide Children’s Hospital, Columbus, Ohio; ^3^Division of Allergy and Immunology, The Abigail Wexner Research Institute at Nationwide Children’s Hospital, Columbus, Ohio; ^4^Department of Pediatrics, The Ohio State University, Columbus, Ohio; ^5^Division of Medical Laboratory Science, Wexner Medical Center, School of Health and Rehabilitation Sciences, The Ohio State University, Columbus, Ohio; ^6^Institute for Behavioral Medicine Research, The Ohio State University, Columbus, Ohio; ^7^Department of Microbial Infection and Immunity, The Ohio State University, Columbus, Ohio; ^8^Department of Neuroscience, The Ohio State University, Columbus, Ohio

**Keywords:** asthma, corticosteroid insensitivity, STAT1, Th1 inflammation

## Abstract

Corticosteroid insensitivity in asthma limits the ability to effectively manage severe asthma, which is characterized by persistent airway inflammation, airway hyperresponsiveness (AHR), and airflow obstruction despite corticosteroid treatment. Recent reports indicate that corticosteroid insensitivity is associated with increased interferon-γ (IFN-γ) levels and T-helper (Th) 1 lymphocyte infiltration in severe asthma. Signal transducer and activator of transcription 1 (STAT1) activation by IFN-γ is a key signaling pathway in Th1 inflammation; however, its role in the context of severe allergic airway inflammation and corticosteroid sensitivity remains unclear. In this study, we challenged wild-type (WT) and *Stat1^−/−^* mice with mixed allergens (MA) augmented with c-di-GMP [bis-(3′-5′)-cyclic dimeric guanosine monophosphate], an inducer of Th1 cell infiltration with increased eosinophils, neutrophils, Th1, Th2, and Th17 cells. Compared with WT mice, S*tat1^−/−^* had reduced neutrophils, Th1, and Th17 cell infiltration. To evaluate corticosteroid sensitivity, mice were treated with either vehicle, 1 or 3 mg/kg fluticasone propionate (FP). Corticosteroids significantly reduced eosinophil infiltration and cytokine levels in both c-di-GMP + MA-challenged WT and *Stat1^−/−^* mice. However, histological and functional analyses show that corticosteroids did not reduce airway inflammation, epithelial mucous cell abundance, airway smooth muscle mass, and AHR in c-di-GMP + MA-challenged WT or *Stat1^−/−^* mice. Collectively, our data suggest that increased Th1 inflammation is associated with a decrease in corticosteroid sensitivity. However, increased airway pathology and AHR persist in the absence of STAT1 indicate corticosteroid insensitivity in structural airway cells is a STAT1 independent process.

## INTRODUCTION

Corticosteroids have broad anti-inflammatory effects that contribute to the effective management of asthma symptoms and exacerbations. In contrast to the corticosteroid-sensitive mild asthma, people with severe asthma exhibit greater airway inflammation, remodeling, airway hyperresponsiveness (AHR), and more frequent exacerbations despite treatment with higher doses of corticosteroids (1, 2). Although the mechanisms that modulate corticosteroid sensitivity and asthma severity remain poorly understood, adaptive immune pathways are thought to play a key role (3). T-helper (Th) 2 inflammation has a central role in many of the pathophysiological and structural features of asthma; however, recent studies suggest that a more complex inflammatory milieu may contribute to corticosteroid insensitivity in severe asthma (4). Studies aimed to define phenotypes and endotypes in severe asthma have identified associations between activation of additional non-Th2 pathways, such as Th1 inflammation and corticosteroid insensitivity (3, 5, 6).

Th1 inflammation can be activated upon viral or bacterial infection and is characterized by increased Th1 lymphocyte infiltration and interferon-γ (IFN-γ) levels (7). Studies in children and adults with severe asthma have identified IFN-γ-producing CD4^+^ T lymphocytes in bronchoalveolar lavage samples (8, 9). Although the source that leads to increased Th1 inflammation remains unclear, studies show that patients with severe asthma have increased colonization of pathogenic bacteria in their airways (10). These observations are supported by studies showing enhanced allergic airway inflammation and corticosteroid insensitivity in mice with Th1 inflammation driven by adoptive transfer of IFN-γ-producing Th1 lymphocytes, administration of lipopolysaccharide or the bacterial second messenger, c-di-GMP [bis-(3′-5′)-cyclic dimeric guanosine monophosphate] (8, 11–13). Furthermore, genetic and pharmacological disruption of IFN-γ signaling has been shown to reduce AHR and airway remodeling in mice (8, 11), implicating Th1 inflammation in functional and structural changes in severe asthma. Although Th1 inflammation has been associated with corticosteroid insensitivity in asthma, it remains unclear how Th1-related signaling pathways affect corticosteroid sensitivity.

Signal transducer and activation of transcription factor (STAT1) is a transcription factor that is activated by IFN-γ and mediates proinflammatory responses in immune and airway structural cells (14). Given the critical role for STAT1 in Th1 lymphocyte differentiation (7), *Stat1^−^*^/^*^−^* mice exhibit reduced Th1 responses and IFN-γ production (15, 16). Previous studies demonstrated that STAT1 phosphorylation induced by IFN-γ remained enhanced in the presence of corticosteroid treatment in vitro (6, 17). Given the potential role for Th1 inflammation in severe asthma, it is important to understand the role of STAT1 in the context of corticosteroid sensitivity.

In this study, we examined corticosteroid sensitivity in wild-type (WT) and *Stat1^−^*^/^*^−^* mice using a chronic mixed allergen (MA) model augmented with c-di-GMP. We hypothesized that ablation of STAT1 would disrupt Th1 inflammation and increase corticosteroid sensitivity in chronic severe allergic airway inflammation. However, our data demonstrate reduced corticosteroid sensitivity in both WT and *Stat1^−^*^/^*^−^* mice. Examination of immunological, functional, and structural aspects of chronic allergic airway inflammation shows that persistent airway remodeling remains increased in *Stat1^−^*^/^*^−^* mice despite treatment with corticosteroids. Our studies demonstrate that corticosteroid insensitivity remains in the absence of STAT1 signaling during severe allergic airway inflammation.

## MATERIALS AND METHODS

### Mouse Allergen Sensitization and Challenge

**Figure 1.**
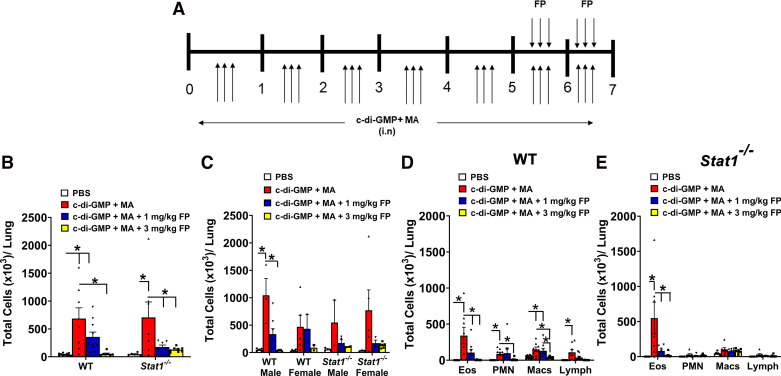
Corticosteroids reduce total airway immune cell and eosinophilic infiltration. *A*: mice were challenged intranasally (in) with c-di-GMP and mixed allergen (MA) 3 times a week for 7 wk. In addition, mice were treated with either vehicle or 1 or 3 mg/kg fluticasone propionate (FP) 3 times a week during *weeks 6* and *7*. *B*: immune cell infiltration in bronchoalveolar lavage (BAL) is increased in both c-di-GMP + MA-challenged wild-type (WT) and *Stat1^−^*^/^*^−^* mice. Treatment with 1 mg/kg FP reduced total BAL cells in c-di-GMP + MA-challenged *Stat1^−^*^/^*^−^* mice. *n* = 6–11 mice per group. *C*: comparison of BAL immune cell infiltration between male and female WT and *Stat1^−^*^/^*^−^* mice. Treatment with 3 mg/kg FP reduced total BAL cells in allergen-challenged male and female mice. *n* = 2–8 male and female mice per group. *D*: differential cell counts show that treatment with 1 and 3 mg/kg FP significantly reduces BAL eosinophil, neutrophil, and macrophage infiltration in WT mice. *E*: treatment with 1 and 3 mg/kg FP significantly reduces eosinophilic infiltration in c-di-GMP + MA-challenged *Stat1^−^*^/^*^−^* mice. Male and female mice were analyzed separately to assess sex-related differences. Data are presented as means ± SE, *n* = 6–11 mice per group, *P* < 0.05. *Significant difference.

Mouse studies were approved by the Nationwide Children’s Hospital Institutional Animal Care and Use Committee. Wild-type C57BL/6J (Stock No. 000664) and *Stat1^−^*^/^*^−^* (Stock No. 012606) mice were procured from Jackson Laboratory (Bar Harbor, ME). Mice were maintained in Research Building III at the Abigail Wexner Research Institute and provided food and water ad libitum. Adult male and female mice were mated to generate pup litters that were randomly assigned a treatment group. Newborn male and female mice were intranasally (in) sensitized and challenged with PBS or mixed allergens (MA), which consists of 10 µg *Alternaria alternata* (Stallergenes Greer, Lenoir, NC), 10 µg *Aspergillus fumigatus* (Stallergenes Greer), 10 µg *Dermatophagoides pteronyssinus* (Stallergenes Greer), and 10 µg ovalbumin (OVA) plus 0.5 µg c-di-GMP (GMP; InvivoGen), three times per week for 7 wk. During the last 2 wk, mice were intraperitoneally (ip) injected with vehicle (PBS containing 0.015% DMSO) or 1 mg/kg or 3 mg/kg FP (FP; Cat. No. 20703; Cayman Chemical, Ann Arbor, Michigan) on the same day as MA challenge. Necropsy was performed ∼24 h after last allergen challenge (Fig. 1*A*). Mice were euthanized by intraperitoneal injection with 90–120 mg/kg ketamine and 5 mg/kg xylazine.

### Bronchoalveolar Lavage Harvesting

Bronchoalveolar lavage fluid (BAL) was harvested as previously described (18). Briefly, whole lungs were lavaged with PBS three times. Collected BALF was centrifuged at 300 *g* for 5 min. Supernatant was collected and stored at −80°C for further analyses. Cellular pellets were resuspended in PBS for total and differential cell count analysis. Total counts were performed using a hemocytometer. Cytospins were generated on microscope slides and differentially stained using a modified Wright-Giemsa Stain (Newcomer Supply, 9112B, Middleton, WI). Cells were imaged on a bright-field microscope and counted by a blinded investigator until a total of 200 cells had been counted.

### Lung Function

Once anesthetized, the trachea was exposed and cannulated with a 19-gauge blunt tip cannula. The mouse was placed on a 37°C heating pad and attached to Y-tubing on the Flexivent (SCIREQ, Montreal, Quebec, Canada). A series of measurements including Snapshot (resistance, compliance, elastance) maneuvers were performed following nebulization with PBS, 6.25, 12.5, 25, and 50 mg/mL methacholine (Millipore-Sigma, St. Louis, MO). Airway hyperresponsiveness is reported as total resistance in response to methacholine.

### Cytokine Analyses

Lung tissue was homogenized in RIPA buffer, processed, and protein concentration measured using Bradford assay. Each well in custom Meso scale U-Plex Multiplex ELISA plates (Meso Scale Discovery, Rockville, MD) was coated with antibodies against IL-4, IL-17A, and IFN-γ. Analytes were measured in whole lung homogenates following manufacturer instructions. IL-13 levels in BAL were measured using ELISA (Cat. No. M1300CB, R&D Systems, Minneapolis, MN) according to the manufacturer’s instructions.

### Western Blotting

Samples were quantified by BCA assay (Pierce) and 25 µg of protein was analyzed per sample. Samples were separated on a 14% SDS-PAGE gel and transferred to nitrocellulose membrane (Bio-Rad). Membranes were blocked for 1 h at room temperature in 1% milk/TBS-T and then incubated in primary antibody overnight at 4°C. Primary antibodies were purchased from Cell Signaling (Danvers, MA): monoclonal rabbit anti-Stat1 antibody at 1:1,000 dilution, Cat. No. 14994S; polyclonal rabbit antiphospho-Stat1 (Ser727) antibody at 1:500 dilution, Cat. No. 9177S; monoclonal rabbit anti-Stat6 at 1:500, Cat. No. 5397S; and monoclonal rabbit anti-phospho-Stat6 (Tyr641) at 1:500, Cat. No. 56554S. The following day, samples were washed in Tris-buffered saline-Tween 20 (TBST), incubated with secondary antibodies (donkey anti-rabbit 800CW or donkey anti-mouse 680RD, 1:20,000, LI-COR) for 1 h at room temperature, washed with TBST, and imaged on an Odyssey Clx (LI-COR). Protein quantification was performed using ImageStudio Software (LI-COR).

### Histopathological Analyses

Left lung lobes were inflated with 10% neutral-buffered formalin at 25 cm H_2_O. Lungs were processed, paraffin embedded, cut into 6 µm sections, and stained with hematoxylin and eosin (H&E, pathological assessment). H&E slides were analyzed and scored for inflammation (0–4 scale) and bronchial-associated lymphoid tissues (BALTs) were quantified by a blinded investigator. Inflammation scores are based on the degree of immune cell infiltration/aggregation around peribronchiolar and perivascular spaces. To quantify BALTs, compact lymphoid nodules were counted in proximal and distal regions. To quantify abundance of mucous cells in the airway epithelium, left lung lobe sections were stained with Alcian Blue-Periodic Acid Schiff (AB-PAS) and photomicrographs of four different airways were taken at ×100 using an Olympus BX-40 light microscope and digital camera (Olympus, Center Valley, PA). PAS-positive and -negative cells were quantified by a blinded investigator using ImageJ (NIH) and reported as a percentage of total airway epithelial cells counted.

### Immunohistochemical Analyses for α-Smooth Muscle Actin

Formalin-fixed, paraffin embedded left lung lobe sections (6 µm) were used for immunohistochemical staining for α-smooth muscle actin (α-SMA). Sections were deparaffinized with xylene (2 × 5 min each) and rehydrated with graded ethanol (100%, 90%, 70%, and tap water). Antigen retrieval was performed with 10 mM sodium citrate at 100°C for 1 h. The slides were blocked for 1 h in TBS containing 4% goat serum and 0.04% Triton X-100. After washing, slides were incubated overnight in monoclonal mouse anti-α-SMA at 1:100 dilution (Millipore Sigma, St. Louis, MO, Cat. No. A2547). After incubation, slides were washed and incubated with anti-mouse-FITC secondary antibody (Millipore Sigma, Cat. No. F0257) at 1:1,000 dilution. Negative control slides followed the same protocol but without addition of a primary antibody. Slides were counterstained with DAPI, and images taken at ×100 using Lionheart (Biotek, Winooski, VT). Smooth muscle actin area was analyzed using ImageJ (NIH). Data were normalized to length of airway basement membrane to report as airway smooth muscle (ASM) mass per µm basement membrane.

### Gene Expression Analyses

RNA (300–1000 ng) from profiled samples was cDNA transcribed using Oligo dT_12–18_ primers and Superscript IV (Thermo Fisher Scientific Applied Biosystems). Quantitative real-time PCR with TaqMan using Muc5ac (Mm0127618_m1), Muc5b (Mm00466391_m1), and GAPDH (Mm99999915_g1) primer sets was performed (Life Technologies) according to the manufacturer’s instructions. Samples were run on a QuantStudio 3 96-well Real-Time PCR system (Thermo Fisher Scientific Applied Biosystems). Results were analyzed using the comparative Ct method for TaqMan assays.

### Flow Cytometry

Lungs were perfused with 5 mL of PBS through right ventricle of the heart, and then placed in C-tubes (Miltenyi Biotec, Gladbach, Germany) and digested for 30 min in 432 U/mL Collagenase Type IV (Worthington Biochemical, Lakewood, NJ, Cat. No. LS004188) and 64 U/mL DNAase I (Millipore Sigma, Cat. No. DN25-100MG) in DMEM medium. Lung tissue was homogenized using Gentlemacs octo dissociator (Miltenyi Biotec), strained through 70 μm nylon cell strainer, centrifuged at 300 *g* for 10 min, and resuspended in 10 mL of DMEM medium. Cells were counted using a hemocytometer. For Th stimulation, two million cells were incubated in DMEM medium containing leukocyte activation cocktail with Golgi plug (BD Biosciences, Cat. No. 550583), protein transported inhibitor containing Monensin (BD Biosciences, Cat. No. 554724), and 10% FBS for 37°C for 4 h. Cells were collected and permeabilized/fixed using fixation/permeabilization buffer and 1× permeabilization/wash buffer (BD Biosciences, Cat. No. 554714) and resuspended in 200 μL of PBS. Single cell suspensions were used in flow cytometry analysis.

Single cell suspensions were stained with various fluorochrome conjugated anti-mouse monoclonal antibodies for cellular phenotyping as follows: FITC-conjugated anti-CD45 (30-F11), BV421-conjugated anti-CD3 (17A2), PE-conjugated anti-CD4 (A161A1), Alexa 700-conjugated anti-IFNγ (XMG1.2), APC-conjugated anti-IL-4 (11B11), and APC-conjugated anti-IL-17A (TC11-18H10.1). To control for intracellular cytokine staining, APC (RTK2071) and Alexa fluor 700 (RTK2071) IgG_1_ isotypes were used. All antibodies were purchased from BioLegend. Data were acquired using a BD LSRII flow cytometer (BD Biosciences, San Diego, CA) and analyzed using FlowJo software (v. 10.7.1).

### Statistical Analyses

Data were analyzed by performing two-way ANOVA with Bonferroni post hoc analysis for multiple comparisons. Data were analyzed and graphed using GraphPad Prism 9 Software (GraphPad, La Jolla, CA). Values are presented a means ± standard error and significant differences indicated by *P* < 0.05.

## RESULTS

### Corticosteroids Reduce Total Airway Immune Cell and Eosinophil Infiltration

Wild-type (WT) and *Stat1^−^*^/^*^−^* mice challenged with c-di-GMP + MA exhibited increased total BAL immune cells compared with PBS-challenged WT mice (Fig. 1*B*). BAL total cell counts were increased in both male and female mice (Fig. 1*C*). Differential immune cell analyses showed that c-di-GMP + MA-challenged WT mice exhibited significantly increased eosinophil, neutrophil, macrophage, and lymphocyte numbers in BAL (Fig. 1*D*). In contrast, neutrophils, macrophages, and lymphocytes were not increased in c-di-GMP + MA-challenged *Stat1^−^*^/^*^−^* mice (Fig. 1*E*). Treatment with 1 mg/kg fluticasone propionate (FP) significantly reduced total BAL immune cells in c-di-GMP + MA-challenged *Stat1^−^*^/^*^−^* mice, but not WT mice (Fig. 1*B*), whereas treatment with the higher dose of FP, 3 mg/kg, led to reduced total BAL and eosinophil cell numbers in c-di-GMP + MA-challenged WT and *Stat1^−^*^/^*^−^* mice (Fig. 1, *B*, *D*, and *E*). In addition to eosinophils, neutrophil and macrophage cell numbers were significantly reduced in c-di-GMP + MA-challenged WT mice treated with 3 mg/kg FP (Fig. 1, *D* and *E*). Effects of FP were comparable in male and female mice (Fig. 1*C*).

### Genetic Deletion of STAT1 Reduces Th1 and Th17 Lymphocyte Populations and Interferon-γ Expression

**Figure 2.**
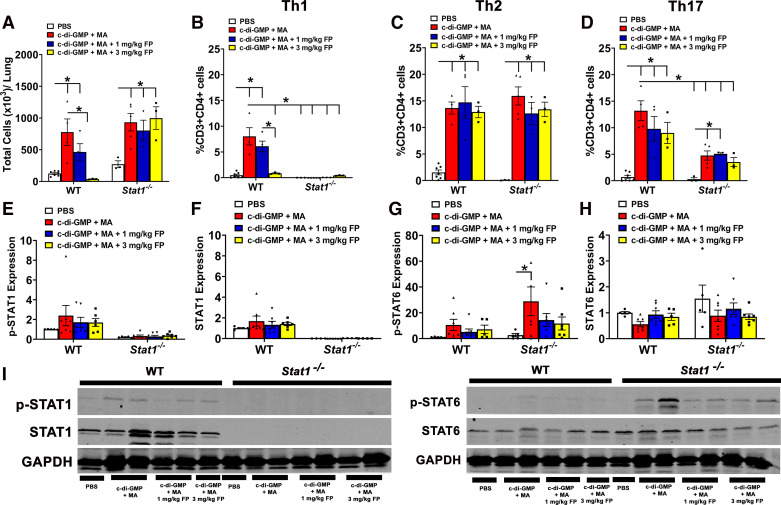
Genetic deletion of signal transducer and activator of transcription 1 (STAT1) reduces T-helper (Th) 1 lymphocyte populations. *A*: total CD3^+^CD4^+^ lymphocytes in wild-type (WT) mice, but not *Stat1^−^*^/^*^−^* mice, are reduced by fluticasone propionate (FP). *B*: Th1 lymphocytes are increased in c-di-GMP + MA-challenged WT mice but reduced in *Stat1^−^*^/^*^−^* mice. In WT mice, treatment with 3 mg/kg FP reduced this increase in Th1 cells. Th2 (*C*) and Th17 (*D*) lymphocytes are significantly increased in c-di-GMP + MA-challenged WT and *Stat1^−^*^/^*^−^* mice and not reduced with treatment with corticosteroids. C-di-GMP + MA-challenged *Stat1^−^*^/^*^−^* mice significantly reduced Th17 lymphocytes compared with WT counterparts. Stat1 phosphorylation (Ser727; *E*) and total protein levels (*F*) remain unchanged in c-di-GMP + MA WT mice, but were absent in *Stat1^−^*^/^*^−^* mice. *G*: Stat6 phosphorylation (Tyr641) is increased in GMP + MA-challenged *Stat1^−^*^/^*^−^* mice compared with PBS controls, whereas *H*: total Stat6 was unchanged in both WT and *Stat1^−^*^/^*^−^* mice. *I*: representative Western blots of phosphorylated (p)-STAT1, total STAT1 protein, p-STAT6, total STAT6 protein, and GAPDH. Data are presented as means ± SE, *n* = 3–7 mice per group, *P* < 0.05. *Significant difference. MA, mixed allergen.

Recent studies have shown evidence of Th1 inflammation in lung-derived samples collected from patients with severe asthma (8). To assess T helper (Th) lymphocyte populations and their respective cytokines, we performed flow cytometric analyses and ELISA on whole lung homogenates, respectively. Total CD3^+^CD4^+^ T cell numbers were significantly increased in c-di-GMP + MA-challenged WT and *Stat1^−^*^/^*^−^* (Fig. 2*A*). Treatment with 3 mg/kg FP significantly reduced total CD3^+^CD4^+^ T cell numbers in WT mice; however, treatment with 1 and 3 mg/kg FP did not reduce total CD3^+^CD4^+^ T cell numbers in *Stat1^−^*^/^*^−^* mice (Fig. 2*A*). For Th1 inflammation, we analyzed for IFN-γ producing CD3^+^CD4^+^ T cells and IFN-γ levels. C-di-GMP + MA-challenged WT mice exhibited significantly increased Th1 cells (Fig. 2*B*) and IFN-γ levels (Table 1). Treatment with 3 mg/kg fluticasone propionate (FP) significantly reduced Th1 populations and IFN-γ levels in c-di-GMP + MA-challenged WT mice (Fig. 2*B* and Table 1). Increased Th1 populations and IFN-γ levels were not observed in c-di-GMP + MA-challenged *Stat1^−^*^/^*^−^* mice (Fig. 2*B* and Table 1). In addition, we did not observe significant changes in STAT1 phosphorylation (Fig. 2, *E* and *I*) or total STAT1 expression (Fig. 2, *F* and *I*) in the c-di-GMP + MA-challenged WT mice. Importantly, STAT1 expression was not detected in *Stat1^−^*^/^*^−^* mice.

**Table 1.** Proinflammatory cytokine levels in chronic model

Genotype	Cytokine	PBS	c-di-GMP + MA	c-di-GMP + MA + 1 mg/kg FP	c-di-GMP + MA + 3 mg/kg FP
Wild type	IFN-γ	0.51 ± 0.04	2.48 ± 0.47*	1.46 ± 0.66	0.633 ± 0.24^$^
	IL-4	0.21 ± 0.10	77.53 ± 17.96*	8.35 ± 3.19^$^	6.28 ± 1.74^$^
	IL-13	2.67 ± 0.77	286.78 ± 91.56*	20.17 ± 10.83	7.64 ± 1.93^$^
	IL-17A	0.05 ± 0.01	226.50 ± 65.91*	74.07 ± 48.28^$^	19.59 ± 10.11^$^
Stat1^−/−^	IFN-γ	0.01 ± 0.02	0.86 ± 0.22	0.53 ± 0.26^%^	0.19 ± 0.12^%^
	IL-4	0.23 ± 0.170	153.80 ± 63.67*	46.84 ± 21.80^$^	29.97 ± 9.08^$^
	IL-13	2.43 ± 0.86	427.42 ± 136.93*	71.71 ± 31.14^$^	42.67 ± 10.04^$^
	IL-17A	0.038 ± 0.03	189.08 ± 86.25	41.55 ± 25.37	14.37 ± 4.98

Data are presented as means ± SE, *n* = 5–10 mice per group, *P* < 0.05. FP, fluticasone propionate; MA, mixed allergen. *Significant difference from PBS wild-type (WT) mice, $significant effect of FP, and %significant difference from c-di-GMP + MA WT mice.

To assess Th2 inflammation, we measured Th2 lymphocytes (CD3^+^CD4^+^IL-4^+^) and IL-4 and IL-13 expression. Th2 lymphocytes were increased in c-di-GMP + MA-challenged WT and *Stat1^−^*^/^*^−^* mice and were not reduced with 1 or 3 mg/kg FP treatment (Fig. 2*C*). WT and *Stat1^−^*^/^*^−^* mice exhibited significant increases in both IL-4 and IL-13 levels (Table 1), whereas treatment with 1 mg/kg FP significantly reduced IL-4 levels in both WT and *Stat1^−^*^/^*^−^* mice challenged with c-di-GMP (Table 1). IL-13 levels were significantly reduced in c-di-GMP + MA WT and *Stat1^−^*^/^*^−^* mice with 3 mg/kg FP (Table 1). Since STAT6 is important for IL-4 and IL-13-mediated signaling (19), we measured expression of STAT6 phosphorylation (Tyr641) in lung homogenates. We found that STAT6 phosphorylation was significantly increased in GMP + MA-challenged *Stat1^−^*^/^*^−^* mice (Fig. 2, *G* and *I*), whereas total Stat6 levels remained unchanged (Fig. 2, *H* and *I*).

In regard to Th17 inflammation, Th17 (CD3^+^CD4^+^IL-17A^+^) cells and IL-17A levels were significantly higher in c-di-GMP + MA-challenged WT and *Stat1^−^*^/^*^−^* mice (Fig. 2*D* and Table 1). Treatment with 3 mg/kg FP significantly reduced IL-17A expression levels; however, Th17 lymphocytes remained increased. There was also a significant reduction of Th17 cell populations in c-di-GMP + MA-challenged *Stat1^−^*^/^*^−^* mice compared with their WT counterparts (Fig. 2*D*).

### Genetic Deletion of STAT1 Does Not Improve Airway Pathology or AHR in Combination with Corticosteroids

**Figure 3.**
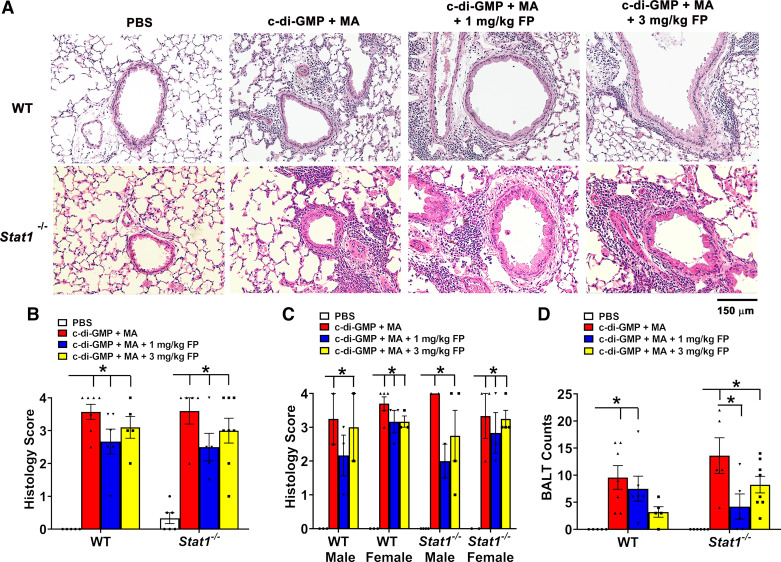
Ablation of signal transducer and activator of transcription 1 (STAT1) does not reduce allergic airway inflammation. *A*: representative photomicrographs of left lung lobe sections stained with hematoxylin and eosin (H&E). *B*: c-di-GMP + MA-challenged wild-type (WT) and *Stat1^−^*^/^*^−^* mice exhibited increased inflammation (increased histology score) in the peribronchiolar and perivascular spaces that was not reduced with corticosteroid treatment. *C*: comparison of airway inflammation between male and female WT and *Stat1^−^*^/^*^−^* mice. *n* = 2–5 male and female mice per group. *D*: number of bronchial-associated lymphoid tissues (BALTs) per lung section in GMP + MA-challenged WT and *Stat1^−^*^/^*^−^* mice were increased compared with PBS controls. Male and female mice were analyzed separately to assess sex-related differences. Data are presented as means ± SE, *n* = 4–8 mice per group, *P* < 0.05. *Significant difference. FP, fluticasone propionate; MA, mixed allergen.

To histologically assess airway inflammation, H&E-stained lung sections were scored for immune cell infiltration in peribronchiolar and perivascular spaces, and bronchial-associated lymphoid tissue (BALTs) nodules quantified. Compared with PBS challenge, c-di-GMP + MA challenge significantly increased airway inflammation in WT and *Stat1^−^*^/^*^−^* mice (Fig. 3, *A* and *B*). These effects were not significantly reduced by treatment with 1 or 3 mg/kg FP (Fig. 3*B*). To analyze for sex-related differences in airway inflammation, male and female WT and *Stat1^−^*^/^*^−^* mice were analyzed separately. C-di-GMP + MA-challenged male and female mice exhibited a significantly higher degree of airway inflammation regardless of genotype. Treatment with 1 and 3 mg/kg FP did not significantly reduce airway inflammation in both male and females regardless of genotype (Fig. 3*C*). In regard to BALTs, c-di-GMP + MA-challenged WT and *Stat1^−^*^/^*^−^* mice exhibited significantly higher number of BALTs in analyzed lung sections compared with PBS-challenge controls (Fig. 3*D*). A significant reduction in BALT counts was only observed in c-di-GMP + MA-challenged *Stat1^−^*^/^*^−^* mice treated with 1 mg/kg FP (Fig. 3*C*).

**Figure 4.**
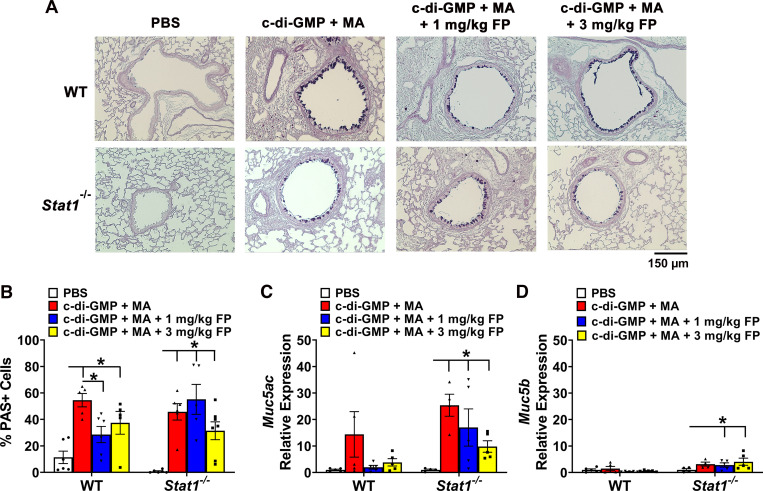
Ablation of signal transducer and activator of transcription 1 (STAT1) does not reduce presence of mucous cells in airway epithelium. *A*: representative photomicrographs of lung sections stained with Alcian Blue-Periodic Acid Schiff (AB-PAS). *B*: allergen-challenged wild-type (WT) and *Stat1^−^*^/^*^−^* mice exhibit increased mucous cell abundance. *Muc5ac* (*C*) and *Muc5b* (*D*) mRNA expression is increased in allergen-challenged *Stat1^−^*^/^*^−^* mice. Data are presented as means ± SE, *n* = 5–9 mice per group, *P* < 0.05. *Significant difference. FP, fluticasone propionate.

To examine the abundance of mucus-producing cells in the airway epithelium, lung sections were stained with AB-PAS and mucous cells counted. Cyclic-di-GMP + MA-challenged WT and *Stat1^−^*^/^*^−^*mice exhibited significant increases in mucous cells compared with PBS-challenged mice (Fig. 4, *A* and *B*). Treatment with 1 mg/kg FP significantly reduced the abundance of mucous cells in c-di-GMP + MA-challenged WT mice (Fig. 4*B*). Mucous cell abundance remained increased in WT and *Stat1^−^*^/^*^−^* mice despite treatment with 3 mg/kg FP (Fig. 4*B*). To determine mucin gene expression, we analyzed whole lung homogenates for expression of secreted mucin-associated genes, *Muc5ac* and *Muc5b* (Fig. 4, *C* and *D*). There were no significant differences in c-di-GMP + MA-challenged WT mice in *Muc5ac* and *Muc5b* mRNA expression. Conversely, c-di-GMP + MA-challenged Stat1*^−^*^/^*^−^* mice exhibited significantly higher *Muc5ac* expression that was not reduced with either FP treatment, whereas c-di-GMP + MA Stat1*^−^*^/^*^−^* mice also had significantly higher *Muc5b* expression compared with PBS-challenged Stat1*^−^*^/^*^−^* mice (Fig. 4, *C* and *D*).

**Figure 5.**
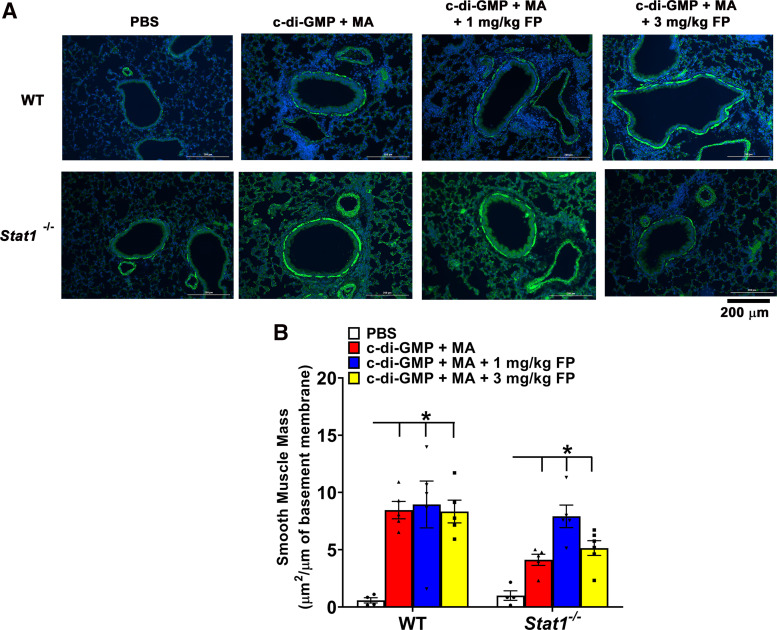
Increased airway smooth muscle (ASM) mass persists in severe allergic airway inflammation. *A*: representative photomicrographs of lung sections immunohistochemically stained for α-smooth muscle actin (α-SMA) and DAPI. *B*: quantification of α-SMA around airways shows increased ASM mass in c-di-GMP + MA-challenged wild-type (WT) and *Stat1^−^*^/^*^−^* mice. These effects were not reduced by treatment with 1 or 3 mg/kg fluticasone propionate (FP). Data are presented as means ± SE, *n* = 4–6 mice per group, *P* < 0.05. *Significant difference. MA, mixed allergen.

To assess airway remodeling, we measured airway smooth muscle (ASM) mass in lung sections stained for α-smooth muscle actin. C-di-GMP + MA-challenged WT and *Stat1^−^*^/^*^−^* mice exhibited greater ASM mass around the airways compared with PBS-challenged mice. The increases in ASM were not reduced in WT and *Stat1^−^*^/^*^−^* mice treated with 1 or 3 mg/kg FP (Fig. 5).

**Figure 6.**
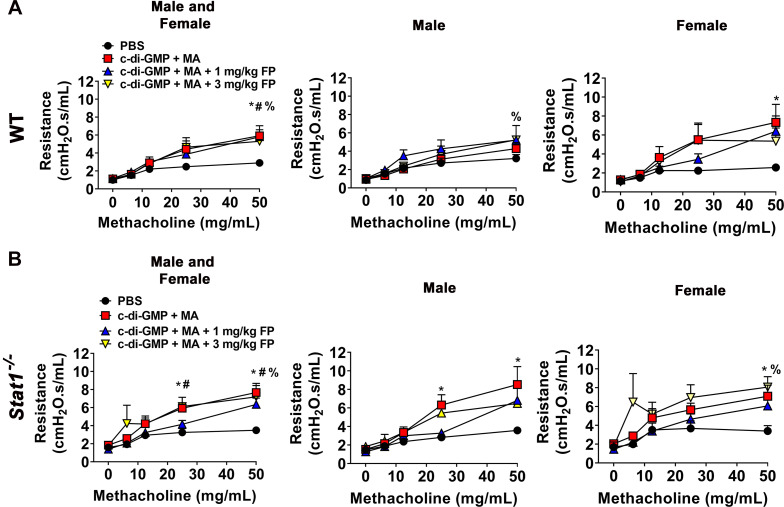
Airway hyperresponsiveness (AHR) in chronic severe allergic inflammation. AHR is significantly increased in mixed allergen (MA) + GMP-challenged wild-type (WT) (*A*) and *Stat1^−^*^/^*^−^* (*B*) mice and is reduced by with the treatment with 1 or 3 mg/kg fluticasone propionate (FP). Male and female mice were analyzed separately to assess sex-related differences. Data are presented as means ± SE, *n* = 8–13 mice per group and *n* = 4–7 male and female mice per group, *P* < 0.05. *, #, %Significant difference from PBS.

Airway hyperresponsiveness (AHR) was measured using forced oscillation maneuvers following administration of methacholine (0–50 mg/mL). C-di-GMP + MA-challenged WT and *Stat1^−^*^/^*^−^* mice showed significantly increased AHR compared with PBS-challenged mice. Treatment with 1 mg or 3 mg/kg fluticasone propionate did not significantly reduce AHR in WT and *Stat1^−^*^/^*^−^* mice (Fig. 6). To analyze for sex-related differences, we next analyzed AHR for males and females separately. C-di-GMP + MA-challenged WT and *Stat1^−^*^/^*^−^* female mice and *Stat1^−^*^/^*^−^* male mice exhibited significantly higher AHR compared with PBS-challenged mice. C-di-GMP + MA-challenged male WT and *Stat1^−^*^/^*^−^* female treated with 3 mg/kg FP also exhibited significantly higher AHR compared with PBS-controls (Fig. 6).

## DISCUSSION

People with severe asthma have a complex inflammatory environment that is thought to contribute to corticosteroid insensitivity (4). Studies suggest that Th1 inflammation promotes corticosteroid insensitivity (4, 8, 17); however, its role remains unclear. Consistent with previous studies, we show that enhanced Th1 inflammation was associated by corticosteroid insensitivity in mice chronically challenged with allergens and a bacterial second messenger (5, 8). C-di-GMP + MA-challenged WT mice exhibited mixed granulocytic cell infiltration with eosinophils and neutrophils, persistent airway inflammation, and dysfunction in airway epithelium and smooth muscle. In addition, Th1, Th2, and Th17 cell populations, as indicated by their respective effector cytokines, were present in our severe allergic airway inflammation model (Table 2). To understand the role of Th1-related pathways in severe allergic airway inflammation, we assessed corticosteroid sensitivity in *Stat1^−^*^/^*^−^* mice that exhibit dysfunctional Th1 signaling (20). Although we hypothesized that *Stat1^−^*^/^*^−^* mice would have improved corticosteroid sensitivity, we found that *Stat1^−^*^/^*^−^* mice remained largely insensitive to corticosteroids. Our data suggest that loss of the Th1-signaling pathway STAT1 does not affect corticosteroid sensitivity and altered airway pathology, inflammation, airway smooth muscle mass, and mucous cell abundance persisted (Table 2).

**Table 2.** Summary of allergen responses and corticosteroid sensitivity in wild-type and Stat1^−^^/^^−^ mice

Endpoint	Effect of c-di-GMP + MA		Corticosteroid Sensitivity	
	Wild Type	*Stat1^−/−^*	Wild Type	*Stat1^−/−^*
Granulocytic infiltration	Eosinophil and neutrophil	Eosinophil	Moderate	Moderate
T Lymphocyte populations	Th1, Th2, Th17	Th2, Th17^lo^	Moderate	Low
Lung inflammation	High	High	Low	Low
Mucous cell abundance	High	High	Low	Low
Airway smooth muscle mass and airway hyperresponsiveness	High	High	Low	Low

MA, mixed allergen; Th, T-helper.

Although BAL immune cell infiltration displayed moderate corticosteroid sensitivity in WT and *Stat1^−^*^/^*^−^* mice, histological analyses showed persistent airway inflammation in both genotypes. Treatment with 1 and 3 mg/kg fluticasone propionate significantly reduced total BAL cell number in WT and *Stat1^−^*^/^*^−^* mice. These decreases were largely attributed to reduced eosinophil infiltration, which is consistent with previous studies in OVA (21) and house dust mite (8) models. In our model, WT but not *Stat1^−^*^/^*^−^* mice had neutrophil infiltration when challenged with c-di-GMP+ MA. Although neutrophils are associated with corticosteroid insensitivity in severe asthma, their infiltration is commonly associated with increased Th17 and not Th1 inflammation (13, 22, 23). Given the role of Th17 inflammation in neutrophil recruitment and lung infiltration (22, 24), reductions in neutrophils in *Stat1^−^*^/^*^−^* mice could be attributed to reduced Th17 lymphocytes and IL-17A expression. STAT1 signaling has been shown to regulate neutrophil infiltration chemoattracts (25, 26), suggesting Th1-associated signaling pathways can contribute to Th17 inflammation and neutrophil recruitment in allergic airway inflammation.

Recent studies led us to hypothesize that disruption of a key Th1 signaling pathway, such as STAT1, would lead to enhanced corticosteroid sensitivity and alleviate severe allergic airway inflammation (5, 6, 8). *Ifn*γ^−/−^ mice challenged with house dust mite (HDM) and c-di-GMP exhibited reduced AHR even in the absence of corticosteroids, implicating Th1 inflammation in increased AHR (8). In addition, these mice showed no significant increase in expression of Th2 cytokines, IL-4, IL-5, and IL-13 (8). In contrast, our studies show that disrupting STAT1 signaling reduced IFN-γ expression and Th1 inflammation and did not lead to reduction in airway inflammation, AHR, or corticosteroid insensitivity. We found evidence that T lymphocyte infiltration and Th2 inflammation maybe enhanced in *Stat1^−^*^/^*^−^* mice. We observed increased CD3^+^CD4^+^ total cell numbers, Th2 lymphocytes, IL-4 and IL-13 levels, and STAT6 phosphorylation in allergen-challenged *Stat1^−^*^/^*^−^* mice. IL-4 and IL-13 are known to have profound effects on airway inflammation, AHR, remodeling, and mucous cell metaplasia, effects that involve STAT6 (19, 27–29). Although we observed that corticosteroids significantly reduced IL-4 and IL-13 expression levels in *Stat1^−^*^/^*^−^* mice, their levels did not return to baseline. IL-4 and IL-13 at relatively low levels have been shown to induce mucus production, AHR, and remodeling (27–30). We speculate that loss of Th1 inflammation within a complex inflammatory environment could allow the effects of Th2 inflammation to continue in the presence of corticosteroids.

Diminished lung function and airway remodeling contribute to airway narrowing and stiffening in severe asthmatics (2, 31). AHR and ASM mass, both key features of asthma, were corticosteroid insensitive in both WT and *Stat1^−^*^/^*^−^* mice. These key indicators of airway structure and function are commonly found to persist despite corticosteroid treatments in severe asthmatics (32, 33). Although it remains unclear why ASM are insensitive to corticosteroids in our models, the continued presence of one or more Th1, Th2, and/or Th17 effector cytokines during airway inflammation may play a role (34). Many of these cytokines are known to increase ASM inflammatory mediator production, hypercontractility, proliferation, and extracellular matrix deposition (30, 34–38). Although corticosteroids are able to reduce the effects of individual cytokines in vitro, the complex inflammatory environment and/or duration of allergen exposure may allow ASM dysfunction to persist. Despite the loss of the Th1 inflammation in *Stat1^−^*^/^*^−^* mice, we observed that AHR and ASM mass remained increased, which is a common feature in patients with severe asthma (2, 4, 33). The severity of airway inflammation was also exemplified by the presence of lymphoid cell aggregations or BALTs in allergen-challenged WT and *Stat1^−^*^/^*^−^* mice. The role of BALTs in asthma is not well understood. However, they are thought to play an important role in localized immune responses in the lung (39, 40) and have been observed in allergen-challenged mice (41–43) and patients with severe asthma (44).

Increased abundance of mucous cells in the airway epithelium is another important pathological feature in severe asthma that also adversely affects airway function by contributing to cough, wheeze, and airway obstruction (45, 46). Previous studies show that the airway epithelium in patients with severe asthma has higher abundance of mucous cells than healthy individuals (27, 46–48). We found significant increases in the abundance of mucous cells in the airways of chronically challenged WT and *Stat1^−^*^/^*^−^* mice. Treatment with 1 mg/kg FP was able to reduce mucous cell abundance in WT mice; however, mucous cell abundance remained increased in *Stat1^−^*^/^*^−^* mice. This observation is consistent with studies demonstrating the inability of corticosteroids to reduce mucous cell abundance in severe allergic airway inflammation (49, 50), making mucous cell metaplasia another important pathologic feature in severe asthma. Increases in mucin gene (*Muc5ac* and *Muc5b*) expression and mucus production are associated with increased Th2-associated inflammation (27). The observed increases in mucin gene expression in allergen-challenged *Stat1^−^*^/^*^−^* mice suggest persistent Th2-associated responses. Collectively, these data show that structural and functional aspects in our model remain largely insensitive to corticosteroids in WT and *Stat1^−^*^/^*^−^* mice.

Our findings in *Stat1^−^*^/^*^−^* mice are in contrast to previous studies in mouse models of severe allergic airway inflammation. In a c-di-GMP + house dust mite (GMP + HDM) model, *Ifn*γ^−/−^ and *Irf5^−^*^/^*^−^* mice exhibited evidence of increased Th2 inflammation with greater eosinophil infiltration than allergen-challenged WT mice (5, 8). However, AHR was significantly reduced in *Ifn*γ^−/−^ and *Irf5^−^*^/^*^−^* mice, whereas we found that airway inflammation and AHR remained increased in *Stat1^−^*^/^*^−^* mice. Although the differences from our study are not entirely clear, our sensitization/challenge strategy, use of fungal allergens (*Aspergillus fumigatus*, *Alternaria alternata*), and persistent airway remodeling could be contributing factors in the lack of reduced airway inflammation and corticosteroid sensitivity in *Stat1^−^*^/^*^−^* mice.

Although Th1 and Th17 inflammation are reduced in *Stat1^−^*^/^*^−^* mice, persistent airway inflammation and corticosteroid insensitivity may involve enhanced Th2 inflammation. Negative regulation of Th2 inflammatory responses by Th1 inflammation is an established and important immune regulatory mechanism (51, 52). This concept is highlighted in studies reporting a role for IFN-γ and STAT1 in suppressing Th2 inflammation. These effects have been attributed to interference with GATA3 expression, Stat6 phosphorylation, and Th2 lymphocyte differentiation (16, 53–55). In the lung, *Stat1^−^*^/^*^−^* mice develop enhanced Th2 inflammation in mouse models of fungal (56) and viral infections (57, 58). In addition, respiratory syncytial virus (RSV) infection has been shown to induce higher levels of IL-13 in the lungs of *Stat1^−^*^/^*^−^* mice compared with their WT counterparts, which lead to increases in mucous cell abundance (57, 58). The antagonistic role for Th1 inflammation in Th2-mediated responses is further demonstrated in recent studies showing that STAT1 is important for limiting ILC2 development and differentiation into effector cells that promote Th2 inflammation (59, 60). ILC2s have an important role in the initiation and maintenance of Th2 inflammation by secreting Th2 cytokines in allergic asthma (61). Persistence of Th2 inflammation may also affect other cells types, such as macrophages and airway structural cells, further contributing to corticosteroid insensitivity in the absence of STAT1.

Sex and circulating sex hormone levels are important factors when considering asthma prevalence. In children, males are much more likely to have asthma than females and to greater severity. As age progresses, females are found to have an increased asthma prevalence compared with males (62). We analyzed our BAL, H&E staining, and AHR to determine potential differences in allergen responses and corticosteroid sensitivity in male and female mice. We did not detect significant differences between males and females in regard to airway inflammation and AHR in WT and *Stat1^−^*^/^*^−^* mice. Despite these observations, little is known about sex differences, corticosteroid sensitivity, and asthma severity. Given the intersections between sex hormone and glucocorticoid signaling in the lung (63), sex is likely an important factor when considering corticosteroid sensitivity. Thus, future studies designed to determine the contribution of sex differences to corticosteroid insensitivity and severe asthma are needed.

Although we found that loss of STAT1 leads to enhancement of STAT6 phosphorylation, we did not examine activation of other pathways associated with Th1- and Th2-mediated signaling in asthma including IRF1, IRF5, and IRS2 (5, 64, 65). Additional limitations to our study include the semiquantitative histological methods used to sample and analyze lung inflammation and ASM mass. Our sampling captured only a portion of airway and lung sections which may not fully represent the effect of allergens and corticosteroids on lung pathology. Cytokine levels, BAL immune cell infiltration, and AHR were not entirely consistent with our histological analyses. Stereological techniques would likely provide more unbiased and comprehensive analysis of lung inflammation and airway remodeling (66). In addition, our understanding of potential sex differences in our model is limited due to relatively low numbers of male and female mice. However, our study does raise intriguing questions about differential corticosteroid sensitivity in males and females with asthma.

In conclusion, our data show that STAT1 is important for Th1 inflammation in severe allergic airway inflammation but not corticosteroid insensitivity. We observed persistent asthmatic features in allergen-challenged *Stat1^−^*^/^*^−^* mice treated with corticosteroids that may involve persistent airway structural alterations such as immune cell aggregation, presence of mucous cells in airway epithelium, and increased smooth muscle mass. These findings may also provide a potential avenue to explore corticosteroid insensitivity in the context of Th2^hi^ severe asthma, which lacks Th1 and Th17 (67). Together, these data provide insight into the role of STAT1 and Th1 inflammation in chronic allergen exposure and has implications for understanding corticosteroid insensitivity in allergic asthma.

## GRANTS

We acknowledge the funding support from National Institutes of Health R00 HL131682 ( to R. D. Britt and D. Jackson), R01 HL155095 (to R. D. Britt), R01 AI121405 (to M. Guerau-de-Arellano), R03 AI151769 (to M. Guerau-de-Arellano), and startup funds from the Abigail Wexner Research Institute at Nationwide Children’s Hospital.

## DISCLOSURES

No conflicts of interest, financial or otherwise, are declared by the authors.

## AUTHOR CONTRIBUTIONS

B.W.L., M.H.G., and R.D.B.J. conceived and designed research; B.W.L., D.J., S.A.A., J.W., M.G., S.G., E.C., A.V.B., and R.D.B.J. performed experiments; B.W.L., D.J., S.A.A., J.W., M.G., S.G., E.C., A.V.B., M.G.-d.-A., and R.D.B.J. analyzed data; B.W.L., M.G.-d.-A., M.H.G., and R.D.B.J. interpreted results of experiments; B.W.L. and R.D.B.J. prepared figures; B.W.L., M.G.-d.-A., M.H.G., and R.D.B.J. drafted manuscript; B.W.L., D.J., S.A.A., J.W., M.G., S.G., E.C., A.V.B., M.G.-d.-A., M.H.G., and R.D.B.J. edited and revised manuscript; B.W.L., D.J., S. A.A., J.W., M.G., S.G., E.C., A.V.B., M.G.-d.-A., M.H.G., and R.D.B.J. approved final version of manuscript.
